# Dynamic Shear Deformation of a Precipitation Hardened Al_0.7_CoCrFeNi Eutectic High-Entropy Alloy Using Hat-Shaped Specimen Geometry

**DOI:** 10.3390/e22040431

**Published:** 2020-04-10

**Authors:** Bharat Gwalani, Tianhao Wang, Abhinav Jagetia, Sindhura Gangireddy, Saideep Muskeri, Sundeep Mukherjee, Jeffrey T. Lloyd, Rajarshi Banerjee, Rajiv S. Mishra

**Affiliations:** 1 Department of Materials Science and Engineering, University of North Texas, Denton, TX 76207, USA; tianhaowang@my.unt.edu (T.W.); abhinavjagetia@my.unt.edu (A.J.); sindhu.g.reddy@gmail.com (S.G.); SaideepMuskeri@my.unt.edu (S.M.); Sundeep.Mukherjee@unt.edu (S.M.); rajarshi.banerjee@unt.edu (R.B.); 2 U.S. Army Research Laboratory, Aberdeen Proving Ground, Aberdeen, MD 21005, USA; Jeffrey.Lloyd@unt.edu; 3 Advanced Materials and Manufacturing Processes Institute, University of North Texas, Denton, TX 76207, USA

**Keywords:** eutectic high-entropy alloy, dynamic shear deformation, split-Hopkinson pressure bar test, hat-shaped specimen, nano-indentation

## Abstract

Lamellar eutectic structure in Al_0.7_CoCrFeNi high-entropy alloy (HEA) is emerging as a promising candidate for structural applications because of its high strength-ductility combination. The alloy consists of a fine-scale lamellar *fcc* + B2 microstructure with high flow stresses > 1300 MPa under quasi-static tensile deformation and >10% ductility. The response to shear loading was not investigated so far. This is the first report on the shear deformation of a eutectic structured HEA and effect of precipitation on shear deformation. A split-Hopkinson pressure bar (SHPB) was used to compress the hat-shaped specimens to study the local dynamic shear response of the alloy. The change in the width of shear bands with respect to precipitation and deformation rates was studied. The precipitation of L1_2_ phase did not delay the formation of adiabatic shear bands (ASB) or affect the ASB width significantly, however, the deformed region around ASB, consisting of high density of twins in *fcc* phase, was reduced from 80 µm to 20 µm in the stronger precipitation strengthened condition. We observe dynamic recrystallization of grains within ASBs and local mechanical response of individual eutectic lamellae before and after shear deformation and within the shear bands was examined using nano-indentation.

## 1. Introduction

Lamellar structures exist widely in various metallic materials such as A356 aluminum alloy (eutectic Al-Si) [1], Mg-Al-Zn magnesium alloy (eutectic Mg-β-Mg_17_Al_12_) [2], pearlitic steel (eutectoid cementite Fe_3_C + ferrite) [3] and Ti-6.5 wt % Si alloy (eutectic Ti-Ti silicide Ti_5_Si_3_) [4] and so on. Generally, a eutectic structure can exhibit high strength and ductility by combining a hard phase and a ductile phase. High-entropy alloys (HEAs) have become a new research frontier in the metallic materials community; they offer a wide range of microstructural tunability from simple single-phase condition to complex eutectic mixtures. Compared to conventional metallic materials, HEAs hold distinct characteristics: (1) high-entropy effect, (2) sluggish diffusion effect, and (3) severe lattice distortion [5,6]. Single-phase HEAs including face-centered cubic (*fcc*), body-centered cubic (*bcc*), hexagonal close-packed (*hcp*), and multi-phase systems including *fcc* + *bcc* phases have been developed [6]. Lu et al. [7] developed a eutectic structured AlCoCrFeNi_2.1_ HEA or EHEA consisting of *fcc* and B2 phases. In this EHEA, high strength of B2 phase and enhanced ductility of *fcc* phase complement each other. Alike AlCoCrFeNi_2.1_, Giwa et al. [8] developed another EHEA based on Al_0.7_CoCrFeNi composition. In our previous work on Al_0.7_CoCrFeNi alloy, we showed [9] that this dual-phase *fcc* and B2 (B2 with *bcc* nano-precipitates) and the alloy strength can be further enhanced via annealing at low temperature by the formation of coherent nano-scale L1_2_ precipitates in the *fcc* phase.

**Figure 1:**
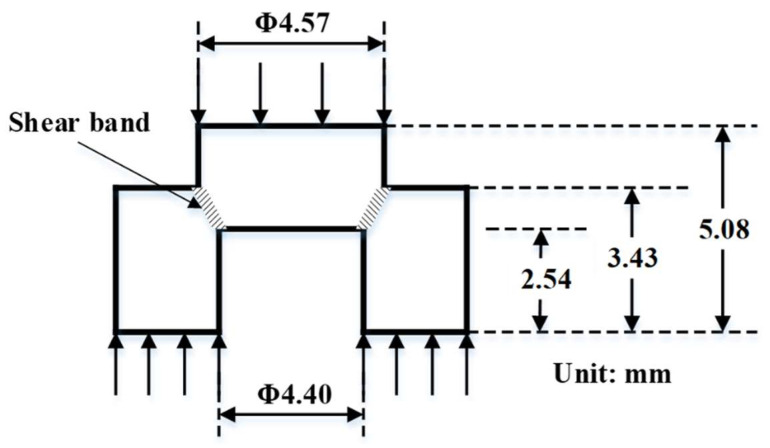
A schematic showing the dimensions of the hat shaped specimen used for shear testing under dynamic loading (10^3^/sec).

Understanding the fundamental mechanism of high strain rate (dynamic) deformation in metallic materials is critical in designing impact-tolerant structures used in automobile, aerospace and defense applications [10]. Dynamic deformation often results in strain localization causing adiabatic shear band (ASB) formation. The thermal softening during ASB formation can lead to premature failure of the component [11]. Therefore, a detailed investigation of the microstructural evolution of ASB in metallic materials before and after impact loading tests is desirable [12,13]. Hat-shaped specimens, commonly referred to as top hat specimens, have been widely used to study the dynamic deformation behavior under shear loading [14,15,16,17,18,19,20,21,22,23,24] using split-Hopkinson pressure bar (SHPB) apparatus. This sample geometry promotes shear localization resulting in deformation focused in a narrow shear band (2–50 µm wide). Inside the shear bands, dynamically recrystallized grains of the order of 20–300 nm in diameter are observed [20], which can exhibit a shear texture. Observation of a wide zone with increasing rotation and elongation of the grains shows an abrupt transition from relatively homogeneous shear. Several important conclusions have been drawn from SHPB tests on hat-shaped specimens of various conventional materials:

(1) Three stages exist during SHPB tests of hat-shaped specimens, namely: (i) the onset of strain localization, (ii) ASB formation, and (iii) micro-cracks initiation and propagation [14]. The width of shear band is narrowed down along the shear direction from top to bottom [15], and its width affects the homogeneity of stress and deformation in shear bands [14]. There is increased recrystallization with strain, which increases from the edge of the shear band and reaches a maximum at the center.
(2) Dynamic recrystallization (DRX) is observed in most of the tested materials including copper [16], steel [17,18], pure titanium [15], titanium alloys [14,18], and aluminum alloy [19]. The shear texture is also observed in low-nickel-containing steel [17], pure titanium [15], and nickel alloy [20]. DRX is confirmed by the emergence of ultra-fined grains with low dislocation density within the shear band [17]. Meyer et al. showed the effect of temperature on shear band width and the extend of dynamic recrystallization in copper [18]. The room temperature shear band microstructure is rather broad (~300 µm) and its thickness is reduced to ~50 µm at 523 K [16]. Wang et al. showed that the grain boundaries in the shear band are geometrical necessary boundaries with high angles. The temperature in shear band is about 943 K which is high enough to meet the thermal needs of recrystallization.
(3) Twinning is an important mechanism for dynamic deformation [15] in medium and low stacking fault energy alloys such as CoCrNi (medium entropy alloy, MEA) and Al_0.3_CoCrFeNi (HEA). Excellent strain hardening in Al_0.3_CoCrFeNi helped in avoiding ASB after SHPB tests of hat-shaped specimens [21].
(4) The geometry of the specimen is key in restricting the deformation mode to shear. The major principal stress σ_I_ is compressive in nature initially. The evolution of hydrostatic stress from compressive to tensile is dictated by the ratio r1/r2 (top radius and bottom radius, refer Figure 1. The overlap between the top and bottom dictates how much the material is under compression initially before it undergoes shear. Peirs et al. showed that the lesser the ratio r1/r2 is from one (the outer diameter of the hat is larger than the inner diameter of the hole in the brim) the higher the force needed to deform the specimen [14]. When r1/r2 = 0.975, the shear stress is homogeneous. When r1/r2 is smaller, hydrostatic pressure becomes much more important. For a very small ratio r1/r2 the experiment is closer to a compression test than a shear test. For specimens with r1/r2 > 0.975, the hydrostatic stress is lower, but the shear stress is less homogeneous along the shear line. Consequently, the calculated average shear stress is not representative for actual shear stress in the center of the shear zone. A specimen with the outer diameter of the hat slightly larger than the inner diameter of the brim is the best compromise between good homogeneity of the shear stress, measurability of the shear stress and a stress state as close as possible to pure shear.

The current work is the first investigation of shear deformation in a eutectic HEAs (EHEAs) and effect of precipitation strengthening on the formation of ASBs in any alloy. The *fcc* phase in the lamellar two-phase microstructure is strengthened by formation of ordered L1_2_ nano-precipitates by an additional low temperature aging, without compromising the tensile ductility of the alloy. We used gun pressures ranging from 10 psi to 40 psi to shoot the Kolsky bar in the SHPB apparatus resulting in increasing displacement rate (velocity of deformation). The width of the ASB increased as the gun pressure was increased. The precipitation of L1_2_ phase did not delay the formation of ASB or affect the ASB width, however, the deformed region around ASB, consisting of high density of twinning in *fcc* phase, was reduced from 80 µm to 20 µm in the stronger precipitation strengthened condition. Nano-indentation results showed the change in hardness of the *fcc* and B2 phases before and after deformation and variation of hardness across the ASB.

## 2. Experimental Section

The Al_0.7_CoCrFeNi alloy (15 at.% Al and 21.25 at.% each of Co, Cr, Fe, Ni) was arc-melted. The ingot was remelted five times for homogenization of the alloy, inverting it after each melt. The as-cast alloy was homogenized at 1150 ^°^C for 1 h to annihilate the dislocations and reduce micro-segregations from casting process to assist in cold deformation of the alloy. The alloy was then rolled at room temperature to 30% reduction in thickness. Subsequent annealing at 1100 for 5 min was done to homogenize and recrystallize the alloy at high temperature. The cold-rolled and homogenized condition is referred to as HTA (high temperature annealed) hereafter. Another low-temperature annealing treatment was done at 580 °C for 24 h on the HTA condition to precipitate a fine-scale distribution of L1_2_ phase inside *fcc* lamellae, this is referred to as the HTA-580 condition. In all the heating steps, the samples were placed in the furnace after the set temperature was reached and cooled by water quenching after a specified treatment time.

The microstructural examination was carried out using scanning electron microscopy (SEM) including electron backscatter diffraction (EBSD) and transmission electron microscopy (TEM). Microstructural characterization was performed using FEI Nova-NanoSEM 230™ coupled with energy dispersive spectroscopy (EDS). Conventional TEM studies were carried out with an FEI Tecnai G2 TF20™ operating at 200 kV. Precipitate characterization was done using conventional and high angle annular dark field-scanning scanning TEM (HAADF-STEM) modes. TEM foils were prepared by an FEI Nova Nanolab 200 dual-beam focused ion beam (FIB) instrument using a Ga ion beam for milling. The ion beam thinning of the samples was done in multiple steps starting from 30 kV ions and finishing with 5 kV ions to reduce the surface damage caused by higher energy ions. Samples were subjected to quasi-static tensile test using the mini tensile testing machine at a strain rate of 1 × 10^−3^ s^−1^ and dynamic deformation using a SHPB apparatus. An emitter working pressure range of 10–40 psi was used to obtain different deformation rates and total strain while maintaining the striker bar length and loading duration constant. The dimensional details of the hat-shaped specimen are presented in the results section in Figure 1. HTA specimens were tested with 20, 30 and 40 psi and HTA-580 specimens were tested with 10 and 20 psi gun pressures. The different gun pressure correspond to different striker bar velocity i.e., 10 psi—7 m/s, 20 psi—11.26 m/s, 30 psi—14 m/s, and 40 psi—17.1 m/s. Different gun pressures were used to assess the effect of deformation rate whereas constant gun pressure of 20 psi on HTA and HTA-580 was used to evaluate the influence of precipitation strengthening on shear deformation. Vickers hardness test was performed on the samples using a load of 500 g for 10 s at regular intervals in a straight line across the shear band. Nano-indentation experiments were performed using TI-Premier Triboindenter (Bruker, Minneapolis, MN, USA) with a diamond Berkovich tip across the deformed and un-deformed regions at a maximum load of 1000 μN. For each indent, the load was linearly increased to 1000 μN in 0.25 s, held constant for 0.25 s, and unloaded in 0.25 s. A spacing of 100 nm was maintained between neighboring indents to avoid overlap of their plastic zones.

## 3. Results and Discussion

### 3.1. Isothermal Aging for Precipitation Strengthening

**Figure 2:**
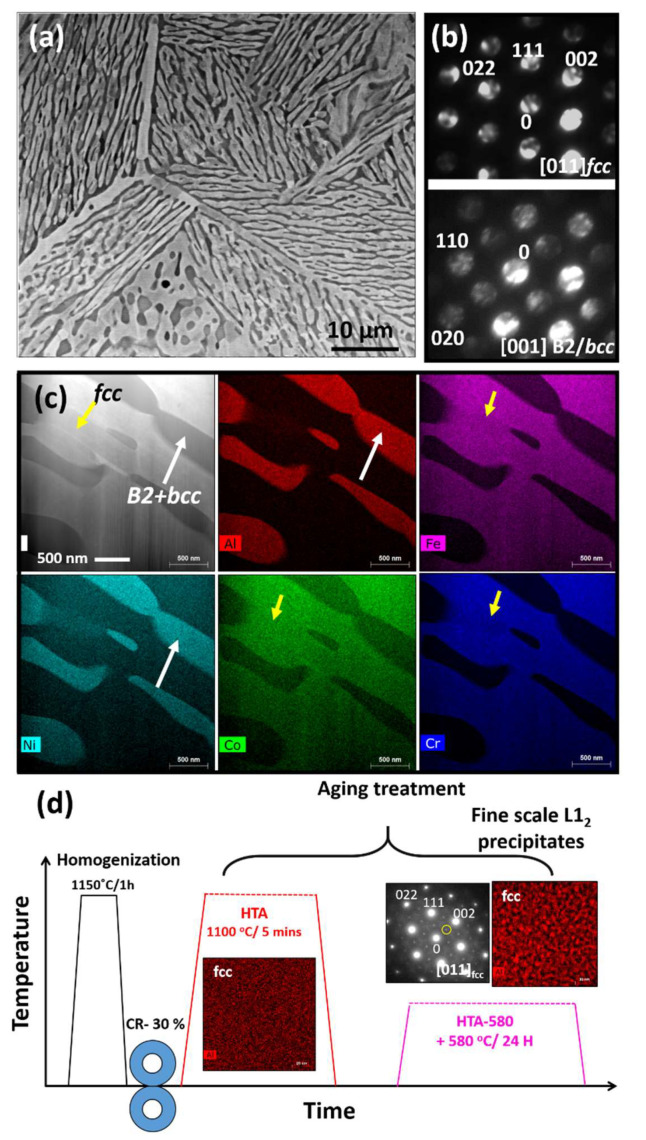
SEM results: (**a**) SEM of high temperature annealed (HTA) condition of Al_0.7_CoCrFeNi alloy; TEM results: (**b**) diffraction pattern from fcc (top) and B2/bcc (bottom) phases; (**c**) energy dispersive spectroscopy (EDS) maps of various elements showing the compositional distribution within the microstructure. Note that the fcc phase (labelled by a yellow arrow in (c) is enriched in Fe, Cr and Co and B2/bcc phase (labelled by a white arrow in (c) is enriched in Al and Ni; and (**d**) schematic of thermo-mechanical treatment to obtain the two different microstructures and high magnification STEM EDS map of Al showing the presence of L1_2_ precipitates in the *fcc* phase after aging treatment at 580 °C.

Al_0.7_CoCrFeNi alloy consists of a lamellar eutectic structure of *fcc* and B2/*bcc* phases (Figure 2). The SEM microstructure from the HTA condition is shown in Figure 2a. The backscattered electron diffraction (BSED) image in Figure 2a shows a two-phase eutectic type of microstructure with a bright contrast phase and dark contrast phase organized in the lamellar arrangement. TEM was used to identify the crystal structure and the composition of the two lamellae. Figure 2b shows the selected area diffraction patterns (SADPs) from the two phases. The SADPs was taken from the bright region in the SEM image was consistently indexed to be a *fcc* phase aligned in [011] zone axis, while the dark region was indexed as *bcc* phase consisting of ordered regions. The [001]***_B2/bcc_*** zone axis is shown in Figure 2 (b, lower part). The presence of extra-super lattice spots in (001) positions in the SADP from [001]***_B2_***_/***bcc***_ zone axis clearly establish the ordering of this phase. The STEM results showed that the *fcc* phase is enriched in Co, Cr and Fe and B2/*bcc* phase are enriched in Al and Ni (Figure 2c) marked with yellow and white arrows respectively in the figure. A detailed high-resolution examination of B2 lamellae showed that it consists of fine-scale precipitation of Cr rich *bcc* phase within it (Supplementary Materials Figure S1). As this B2 + *bcc* phase is consistent in both the heat treatment conditions is not a variable in our study, we would call B2 + *bcc* lamellae as B2 lamellae. The second sample condition (HTA-580) was obtained by heat treating the HTA condition further at 580 °C for 24 h as depicted by the schematic in Figure 2d. HTA-580 condition induced nano-scale L1_2_ precipitates in *fcc* lamellae. The change in Al distribution in HTA and HTA-580 as examined by EDS mapping and the SADP from [111]*_fcc_* in HTA-580 in Figure 2d that verify the presence of ordered nano-scale L1_2_ phase in HTA-580. EDS results of HTA and HTA-580 constituting elemental maps of all elements are shown in Supplementary Materials Figures S2 and S3.

### 3.2. Mechanical Properties

#### 3.2.1. Quasi-Static Tensile Test

**Figure 3:**
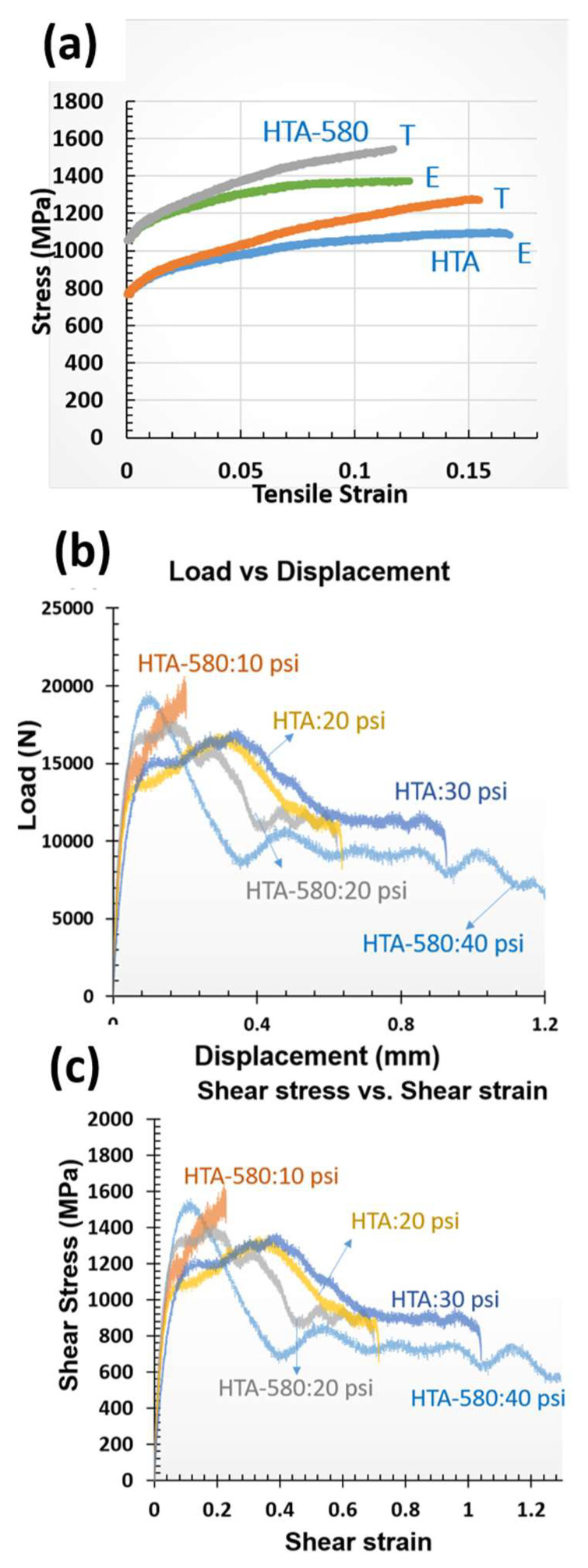
(**a**) Results from the quasi-static tensile testing: stress-strain curves showing the engineering (E) and true (T) stress-strain behavior of HTA and HTA-580 condition; (**b**,**c**) load vs. displacement, and estimated shear stress vs shear strain curves under dynamic compression loading of hat-shaped specimens deformed at different velocities. (10^3^/sec).

Quasi-static tensile tests of both the heat-treated conditions was performed to evaluate the change in mechanical performance due to precipitation of the ordered L1_2_ phase under uniaxial loading. The tensile strength of Al_0.7_CoCrFeNi in HTA-580 condition is higher compared to Al_0.7_CoCrFeNi in HTA condition (Figure 3a). The *fcc* + L1_2_/*bcc* + B2 microstructure (HTA-580) in this alloy exhibited a tensile yield strength (YS) close to 1000 MPa (~990 MPa), ultimate tensile strength (UTS) ~ 1400 MPa and elongation to failure of ~13% while the *fcc*/*bcc* + B2 microstructure in HTA condition showed a YS of 780 MPa, UTS of ~1100 MPa and elongation to failure of 17% under quasi-static loading (strain rate of 10^−3^). The YS increased by 200 MPa which can be attributed to the L1_2_ precipitates in *fcc* lamellae formed after the low-temperature heat treatment at 580 °C. A detailed microstructural examination of post deformed condition after tensile testing can be accessed elsewhere [9].

#### 3.2.2. Dynamic Shear Compression of Top Hat Specimen

In the current work, we also examine the two heat treatment alloy conditions under a localized shear deformation using hat shaped specimens. Gun pressure directly related to deformation velocities ranging from 1.6 m/s (10 psi) to 2.3 m/s (40 psi). Load versus displacement curves for the two heat treatment conditions viz. HTA (*fcc* and B2 + bcc) and HTA-580 (*fcc* + L1_2_ and B2 + bcc) are shown in Figure 3b. The shear stress is estimated by dividing the load by deformed area [π*(h_2_-h_1_) * (r_1_ + r_2_)] and shear strain is estimated by dividing displacement by width (h_2_-h_1_) and are plotted in Figure 3c. The flow stress increases for lower strains as the alloy work hardens, a sudden drop in the flow stress indicates the formation of ASB. Figure 3b,c show the test results from HTA-580 tested with 10 psi and 20 psi pressure and HTA tested with 20 psi, 30 psi and 40 psi gun pressure. The drop in the flow stress is not seen in for 10 psi condition as the net strain is not sufficient to cause the formation of ASB and resulting thermal softening. In all the other conditions we observe a sudden drop in the flow stress which corresponds to the observation of ASB and thermal softening (to be discussed in the next section). Dynamic recrystallization accompanied in the process causes pronounced local softening of the material, while being driven by mechanical strain rather than temperature [24]. Gun pressure of 40 psi resulted in the fracture of the specimen. Note that change in gun pressure leads to the change in velocity of deformation (deformation rate) resulting in change in total strain in the specimen as the time of loading is constant for test.

#### 3.2.3. Microscopic Analysis of the Shear Region in the Deformed Hat Shaped Specimen

**Figure 4:**
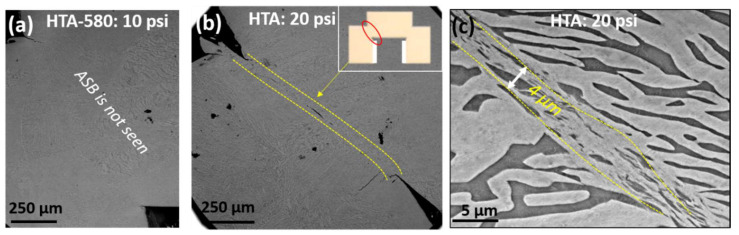

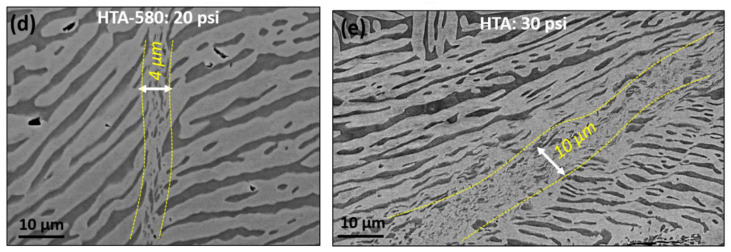
SEM images showing deformed hat-shaped specimen. (**a**) HTA-580 deformed with 10 psi gun pressure, no adiabatic shear band (ASB) is observed. (**b**) HTA deformed using 20 psi, the yellow arrows show the location of cracking and ASB formation. (**c**) high magnification image of the HTA-20 psi condition showing the width of the ABS to be about 4 µm. (**d**) HTA-580:20 psi condition (**e**) HTA:30 psi condition where a 10 µm wide ABS can be seen in the center.

Figure 4a,d shows the SEM images of the cross-section of HTA-580 samples loaded with 10 and 20 psi pressure, respectively, while Figure 4b,c,e presents the deformed region from HTA samples loaded with 20 and 30 psi. Adiabatic shear band is not observed in HTA-580:10 psi condition as the deformation rate is insufficient at this gun pressure to form an ASB. However, as the gun pressure is increased to 20 psi (Figure 4b–d), we see the formation of ASBs. A low magnification SEM image of the cross-section is shown in Figure 4b. The photograph of the cross-section of a deformed hat shaped specimen is shown as an inset and the location of the SEM examination is highlighted by a red circle. The yellow arrow in Figure 4b shows the low magnification image of the ASB formed in HTA: 20 psi condition. The ASB width in this condition is measured to be ~4 µm clearly presented in the high magnification image (Figure 4c,d) shows the BSED image from the HTA-580 condition deformed with 20 psi pressure. The width of the ASB in HTA-580:20 psi is very similar (~4 µm) to that in HTA: 20 psi suggesting no clear influence of precipitation strengthening on ASB formation. However, the width of ASB increased to ~10 µm in HTA: 30 psi specimen, which suggests a direct correlation with the deformation rate and total strain. Higher total strain increased the width of ASB but precipitation strengthening did not have a noticeable influence. In most materials the shear band width (∆) scaled with ∆ = √(KT/(σ*ε)) [25] where K is fraction of plastic work converted to heat, T is temperature, σ is the flow stress and ε is the strain. In our case the shear band width at the onset of localization is similar between the two specimens despite one being stronger than the other. This relationship being known to hold for many materials; however, it may not hold for the case where the length scale of microstructure variations approaches the estimated shear band width. There is insufficient experimental result from the eutectic microstructures on formation of ASBs to conclusively comment on this observation.

**Figure 5:**
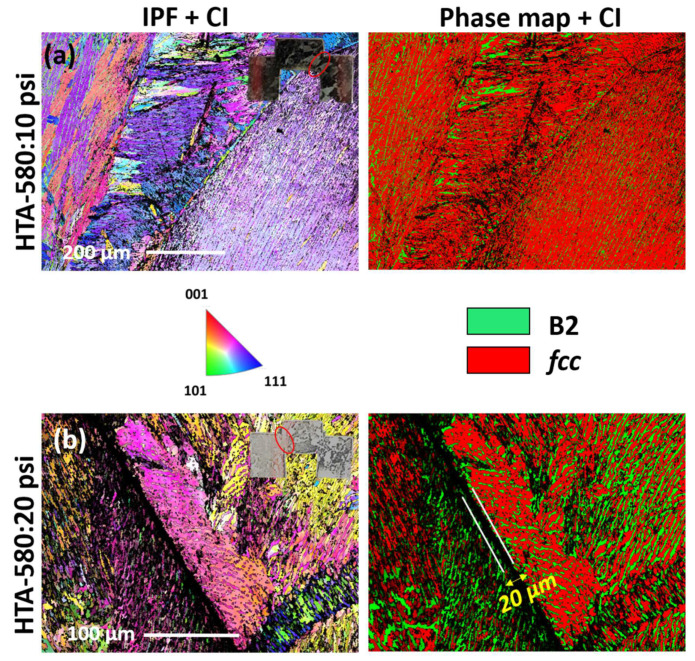
Electron backscatter diffraction (EBSD) inverse pole figures (IPF) and phase map of deformed hat-shaped specimens. (**a**) IPF and phase map from HTA-580: 10 psi. (**b**) IPF and phase map from HTA-580: 20 psi.

**Figure 6:**
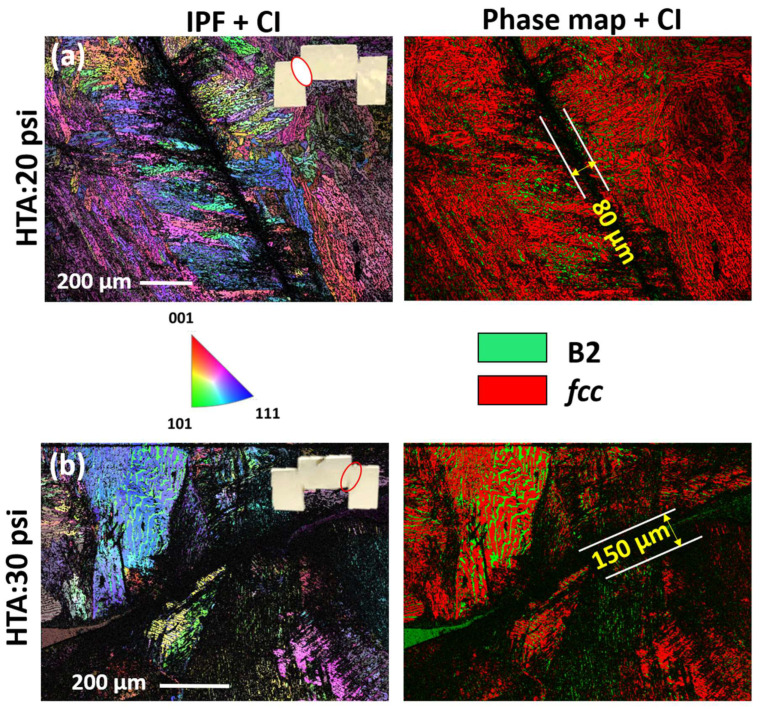
EBSD IPF and phase map of deformed hat-shaped specimens. (**a**) IPF and phase map from HTA: 20 psi. (**b**) IPF and phase map from HTA: 30 psi.

Further analysis of deformed specimens with electron backscattered diffraction (EBSD) orientation image microscopy (OIM) shows that the width of extended deformed region is much larger than the width of ASB observed in the SEM (Figure 5 and Figure 6). The inverse pole figures (IPFs) overlaid with confidence index (CI) and the phase maps overlaid with CI from the different samples and deformation conditions are shown in Figure 5 and Figure 6. Low CI is suggestive of highly deformed structure and hence the width of the highly deformed region is estimated here based on the width of dark contrast (low CI) region in the figure. Figure 5a,b shows the IPF + CI and phase map + CI from HTA-580:10 psi, HTA-580:20 psi, and Figure 6a,b from HTA:20 psi, HTA:30 psi conditions, respectively. Each of the figures has a photograph of the sample cross-section highlighting the region of interest. HTA-580:10 psi did not show any region of low CI which is consistent with SEM images where no ASB formation was observed. The strain hardened and region undergoing plastic deformation around the ASBs are ~80, 20 and 150 µm wide for HTA:20 psi, HTA-580:20 psi and HTA-580:30 psi, respectively, in the deformed specimens. Note, HTA:20 psi and HTA-580:20 psi specimens showed a similar ASB width on SEM-BSED imaging, but the deformed region was much lower for HTA-580:20 psi specimen. Hence, even though the width of ASB only depended on the total strain, HTA-580 condition having *fcc* phase strengthened by coherent L1_2_ precipitates, needed high flow stresses to deform compared to HTA condition. We will now show a detailed TEM examination of the ASBs and the deformed region around it.

**Figure 7:**
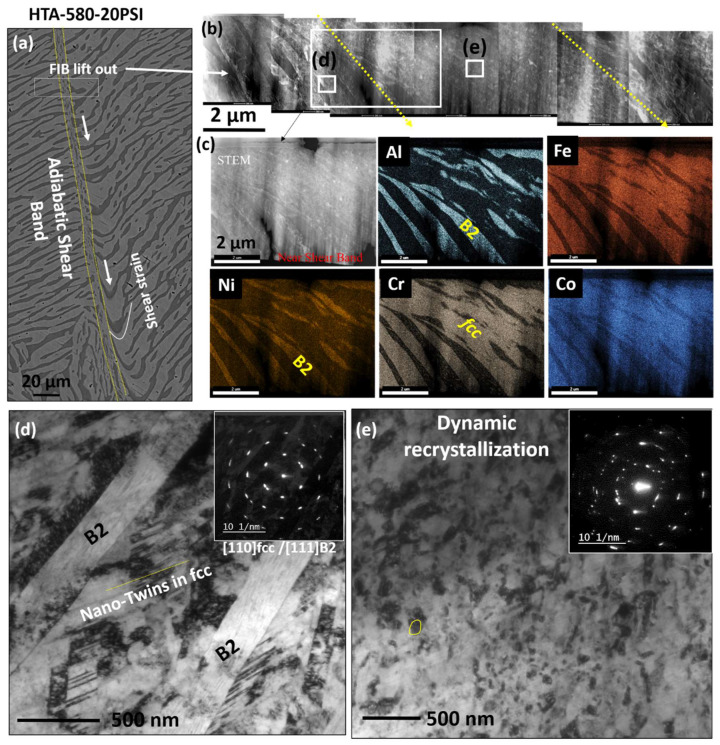
Microstructural analysis on adiabatic shear band in HTA-580:20 psi deformed specimen. (**a**) shows the SEM images highlighting the location of the TEM lift out; (**b**–**e**) show the TEM results; (**b**) shows the STEM image of the TEM foil traversing across the ASB; (**c**) EDS maps from the region highlighted by red rectangle in (**b**); (**d**) BFTEM and the SADP from the blue square in (**b**). This region is just outside the ASB. Note the profuse number of nano-twins and high dislocation density in this region; (**e**) The region inside the ASB marked by yellow box in (**b**). The BFTEM and SADP from this region are shown here. Dynamic recrystallization is observed within the ASB.

Figure 7 shows the TEM results from the ASB region in HTA-580 sample. Figure 7a shows the SEM image highlighting the location of the TEM lift out (white rectangular box). Note that the shear strain results in the bulging of the eutectic lamellae near the ASB in the direction of plastic flow. A montage of STEM images with the image of the overall TEM foil (Figure 7b) shows locations of further analysis by different white boxes. An HAADF STEM image and EDS maps from the region highlighted by a large white box which traverses across the boundary of the shear band are shown in Figure 7c. Note that though we see a fragmentation of the eutectic lamellae within ASB, but the compositional heterogeneity is maintained even within the ASB. Earlier Edwards et al. studied the shear deformation using hat shaped specimen on a 2024 Al alloy [19]. They showed that the high temperature generated with the formation of ASB caused the second phase particles to coalesce and resulted in a slightly smoother dissolution surface on the particles. The smaller particles aligning in the shear flow direction are indicated to form as fragmentation of second phase particles inside the ASB leading to the formation of the rigid walls of the shear band. EDS of a larger second phase particles in their study showed the composition to be different from that of the matrix material [19]. In the current work on examining the region outside the ASB as shown in Figure 7d, we notice that the L1_2_ phase within *fcc* has been disordered and the presence of high density of nano-twins. The BFTEM image in the figure shows a high dislocation density in the region. An SADP pattern from [011]*_fcc_* is shown as the inset on left of the figure, superlattice spots corresponding to the L1_2_ phase are not seen suggesting deformation induced disordering of the matrix. Deformation induced disordering of L1_2_ phase has been reported during severe plastic deformation by Rentenberger et al. [26,27]. They observed that the increase in shear strain resulted in reduction of long-range ordering by forming antiphase boundaries and nucleating nano-crystallization. The yellow lines in the BFTEM image in Figure 7d are highlighting the closely spaced deformation twins in the *fcc* phase. Earlier Kuang et al. found {10–12} < –1011 > tensile and {11–22} < 11 2–3> two types of compressive twins in the deformed region around the shear band in pure Ti after dynamic impact loading [15].

On examining microstructure within an ASB, we noticed nano-structuring of the grains. From Figure 7e, dynamically recrystallized grains of the size scale of 100–200 nm can be noted. One such grain has been highlighted by the yellow color boundary in the figure. The SADP from this region shows a ring-like pattern is shown as the inset, further ascertaining nano-structuring of the grains within the ASB. At high strain rates (> 10^3^ s^−1^), the deformation process is extremely fast and can be considered as an adiabatic process. The temperature inside an ASB is a function of shear strain [22,23]. Meyer et al. showed that the temperature can be ~1000 K in SS304 at a shear strain of 40% [22]. Yang et al. estimated the temperature in pure Ti to be around 1073 K on explosive loading with strain rate of 10^6^/s [23]. The width of shear band in Yang et al. study was 4–8 µm, however, Piers et al. showed that the width depends heavily on the sample geometry and dimensions [14].

Temperature rise in the shear band associated with the deformation plays a significant role in the study of microstructure mechanism and is estimated by the following equation [22,23]:

(1) $$T=T_{0}+\frac{\eta }{\rho C_{v}}\int_{\varepsilon_{s}}^{\varepsilon_{e}}\sigma d\varepsilon$$

where $T_{0}$ is the initial deformation temperature, η is the fraction of plastic energy converted to heat (generally ~90%), ρ is the mass density, $C_{v}$ is the heat capacity, σ is the stress and ε is the strain. For Al_0.7_CoCrFeNi in this study, $T_{0}$ = 293 K, ρ is ~7080 kg/m^3^, $C_{v}$ = 400–550 J/kg·K (C_v_ for steels and nickel based alloys lies between 400–550 J/kg·K), the T can be estimated to be in range of 500 K to 800 K (melting point of the alloy is around 1550 K). Andrade et al. simulated the recrystallization mechanism within a shear band assuming T = 0.5 T_m_ [16] They suggested that the recrystallization happens by grain boundary rotation and limited reorientation. The time taken for the deformation at the strain rate of 10^3^/s in the present study is of the order of a few µsecs. The observation of dynamically recrystallized grains suggest temperature reaching >0.5 T_m_. The localized mechanical response of the individual phases due to shear deformation was evaluated using nano-indentation as detailed below.

#### 3.2.4. Estimation of Instability Strain from Culver Criterion

To predict the strain at which the adiabatic shear band forms, Culver proposed a simple condition for a mechanical instability to begin to form [28,29]. Assuming a pure shear deformation, τ (shear stress) does not increase with γ (shear strain) or d τ /d γ = 0, when we reach the instability strain. Culver derived a simple relation for predicting the instability strain (modified by Staker) [29] γ_i_ = nρC/(dτ/dT) at constant strain rate. γ_i_ is the instability strain, n is strain hardening coefficient, ρ the density of the alloy, C is the specific heat capacity, and T is the temperature. The estimation of dτ/dT is not very straightforward and requires many isothermal experiments. Such a data is rarely available for most alloys. However, this equation implies that a material with large strain hardening coefficient n, should need high instability strain. The value of n and K are measured using the power-law dependency of stress-strain (σ = Kγ^n^). The value of K is 1512 for HTA and 1638 for HTA-580 condition, and n is 0.549 for HTA and 0.512 for HTA-580 (refer Supplementary Materials Figure S5). The density of the Al_0.7_CoCrFeNi alloy is estimated to be ~7300 kg/m^3^ [30] and due to lack of data C is assumed to be equal to SS304 (500 J/kg/°C). The value of (dτ/dT) for 304 steel has been reported to be ~2000 KPa/K [28,29]. Based on these assumptions, the instability strains are roughly estimated to be 1 for HTA and 0.93 for HTA-580. The experimentally observed values are 0.41 for HTA and 0.4 for HTA-580. The increase in instability strain is observed to increase n, however, Culver criteria overestimates the values by a factor of more than two. Such an over estimation was also reported by Walley [28] for titanium alloy. More experimental results are needed to accurately predict the instability strain for the current alloy.

#### 3.2.5. Microhardness Testing and Nanoindentation Details

**Figure 8:**
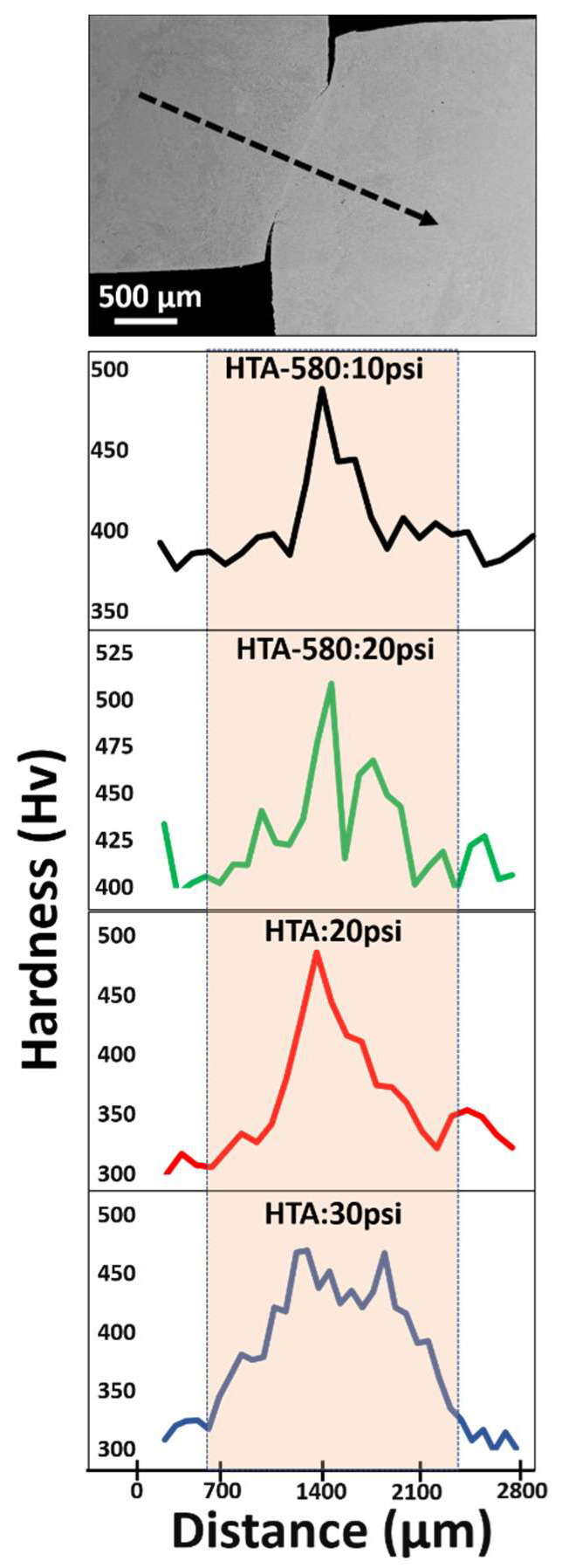
Vickers hardness profile and results for samples under study. Top to bottom- location of indentations, results from HTA-580:10 psi, HTA-580:20 psi, HTA-20 psi, HTA:30 psi conditions respectively.

Microhardness measurements were done on samples HTA:20 psi, HTA-580:10 psi, HTA-580:20 psi and HTA-580:30 psi using a standard Vickers microhardness tester. The indents were spaced at regular intervals of 160–200 µm in a straight line covering approximately 2–3 mm distance (Figure 8). The indent on the shear bands in sample served as a fiducial marker for nano-indents which were performed later in the same region. It was observed that the base hardness in HTA condition was around ~300 Hv while the hardness of HTA-580 sample varied in the range of 370–400 Hv. A hardness vs distance plot is shown in Figure 8 on traversing across the sheared region as shown in the figure by an arrow. An average of three readings was used to plot the curve; it is seen that in all deformation conditions an increase in hardness is seen in the neck region of the top hat specimen where the ASB is formed after deformation. This is due to the strain hardening effects. There a plausible softening effect caused by adiabatic temperature rise inside ASB which was probed using nanoindentation. The width of the spike in hardness in the curves also gives an approximate distance range of plastic deformation in each condition. The spike is broadest in HTA:30 psi and narrowest in HTA:10 psi, which is expected. However, this width is much larger as compared to that noticed on SEM and EBSD characterization.

**Figure 9:**
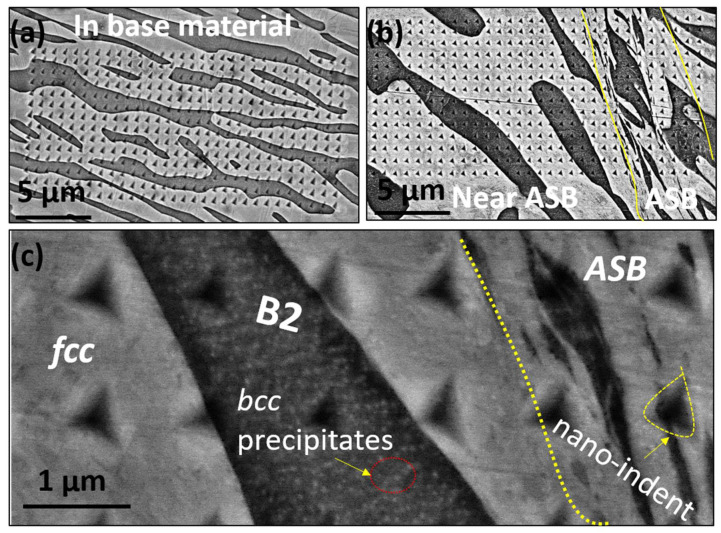
Maps for (**a**) base material of Top Hat specimen; (**b**) map depicting the undeformed, near ASB and ASB; (**c**) high magnification image near shear band showing *fcc* and B2 phases.

Nano-indentation tests were performed using the standard Berkovich tip at the load of 1000 µN to compare the effect of individual phases before and after deformation. Nanoindentation maps of 30 µm × 14 µm were made on the deformed and undeformed region for three top hat samples, namely HTA:20 psi, HTA-580:20 psi and HTA:58030 psi. Figure 9a depicts the map for base material where no deformation was seen. Figure 9b shows the deformed region maps covering the shear band and its surrounding plastic region. The black region is the B2 phase while the white region is the *fcc* phase. A high magnification image covering FCC, B2 phase and ASB is shown in Figure 9c. The nano-indents on B2 and *fcc* phases were identified and approximately 50 indents from the B2 phase and 100 indents from the *fcc* phase were taken for average calculations. Table 1 summarizes the nano-indentation results for various sample conditions. The sample is divided into three regions for characterization viz. in base (away from ASB), near ASB (5–50 µm away from ASB) and within ASB.

The base hardness of *fcc* phase in HTA sample was ~4.2 GPa and that in HTA-580 sample was ~5.2 GPa. The base hardness of the B2 phase in HTA sample was ~6.1 GPa whereas in HTA-580 sample was ~6.9 GPa. Hence, the aging treatment increased the hardness of both the phases. The increase in the hardness of B2 phase is not investigated in the current study. The hardness of *fcc* and B2 phase decreased in both HTA and HTA-580 conditions in near ASB region after 20 psi deformation but it increased for 30 psi deformation. The hardness within ASB was in the range of 3.4–3.7 GPa in HTA and HTA-580 conditions after 20 psi whereas ~5.9 GPa in HTA condition after 30 psi deformation. Vicker’s hardness test, which is a bulk method showed a clear trend of increase in hardness on going from base to deformed region, however, such a trend is not observed in nanoindentation. The anomalous trend in the nano-hardness cannot be explained at this point and needs further study to understand and will be explored in the future. Lastly, we present the microstructural characterization of the failed sample deformed at 40 psi.

#### 3.2.6. Microscopic Analysis of the Fractured Hat-Shaped Specimen

**Figure 10:**
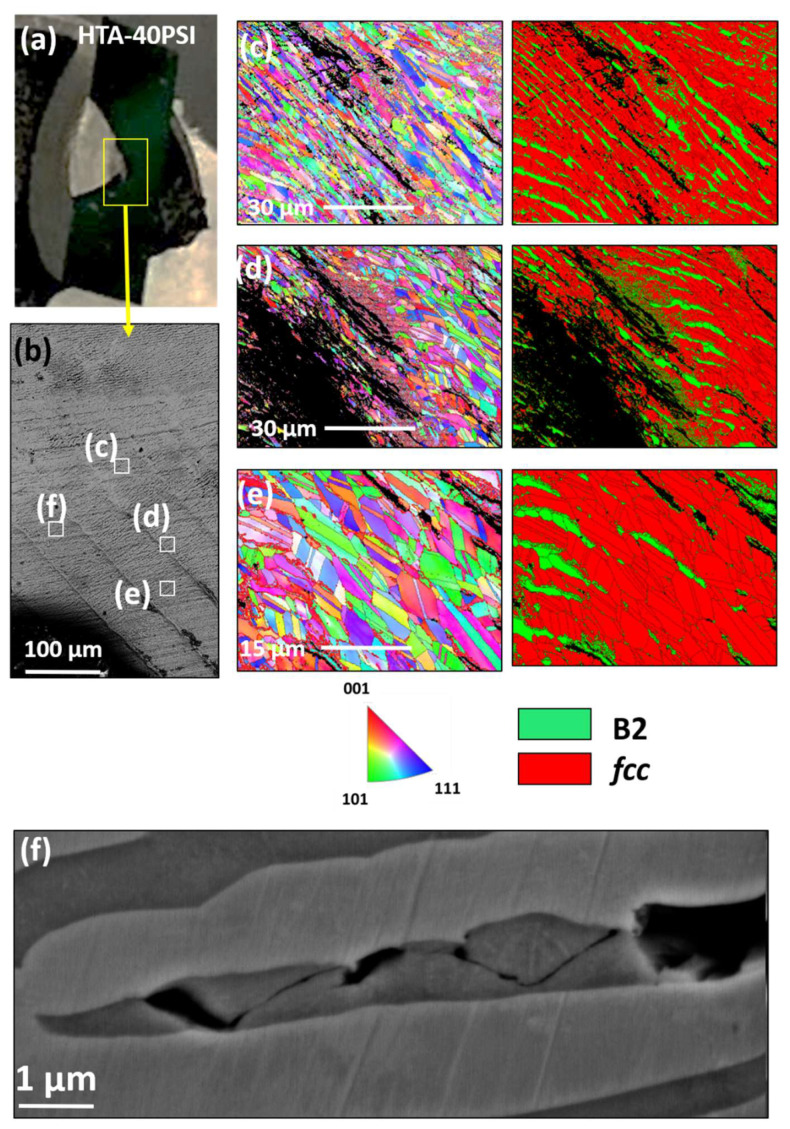
Microstructural analysis on fractured HTA:40 psi specimen: (**a**) photograph of the failed top hat specimen; (**b**) SEM image showing multiple shear band forming in the failed region; (**c**–**e**) EBSD results from the regions highlighted in (**b**); (**f**) SEM showing the crack propagation limited to B2 phase.

Fractured HTA:40 psi specimen shows that the B2 phase is fractured and the SEM examination suggested localized melting of the B2 phase and recrystallization of *fcc* grains (Figure 10 and Supplementary Materials Figure S4). Figure 10a shows the photograph of the fractured sample where a yellow color box marks the region of interest. Figure 10b is an SEM-BSED image showing multiple shear bands and EBSD analysis from specific regions marked with white boxes shown in Figure 10c–e. The IPF and phase maps from each of these regions show that the B2 phase lamellae are fractured and partially melted. Figure 10e clearly shows a highly recrystallized *fcc* grain structure with equiaxed grains and presence of annealing twins. Figure 10f and Supplementary Materials Figure S4 shows that the cracking is limited to the B2 phase and broadening of cracks is seen to accommodate the plastic flow. The temperature estimated by Equation (1) comes out to be ~800 K, however melting of B2 phase suggests localized temperatures reaching around ~1300 K (based on the simulated phase diagram shown in [9]) on loading at 40 psi. Hence, Equation (1) can be underestimating the temperature reached during ASB formation.

## 4. Conclusions

Dynamic shear response of a eutectic HEA (Al_0.7_CoCrFeNi) was investigated via mechanical and microstructural characterization. Hat shaped specimen geometry was used to understand the effect of shear localization in this alloy. A benchmark alloy condition (HTA) consisting of *fcc* and B2 + bcc lamellae were compared with the HTA-580 condition consisting *fcc* + L1_2_ and B2 + bcc lamellae. Aging at 580 °C for 24 h resulted in the formation of nano-scale L1_2_ precipitates in *fcc* lamellae providing precipitation strengthening to the alloy. Dynamic shear testing of the two heat treatment conditions was conducted using a split Hopkinson’s pressure bar apparatus. The two conditions were characterized in detail before and after the dynamic shear loading to comparison of the effect of precipitation strengthening and total strain with respect to adiabatic shear band formation.

The major finding of the paper is summarized as follow:

(1) The tensile yield strength (quasi-static testing at 10^−3^/sec) of the Al_0.7_CoCrFeNi eutectic HEA in HTA condition was 780 MPa which increased to 990 MPa after precipitation of L1_2_ phase by aging at 580 °C for 24 h in the HTA-580 condition. Such an increase in mechanical properties was also noted in nanoindentation response of individual phases (*fcc* and B2) in the HTA-580 condition.
(2) The shear deformation tests were conducted using a split Hopkinson’s pressure bar apparatus. The HTA-580 condition was tested under 10 psi and 20 psi loading conditions and the HTA condition was tested under 20 psi, 30 psi, and 40 psi loading conditions. The 10-psi loading rate (1.6 m/s) was insufficient to cause the formation of ASB whereas the loading rate of 40 psi (2.3 m/s) resulted in the fracture of the specimen. The 20 psi loading rate was used to compare the deformation response in the HTA vs HTA-580 conditions.
(3) Formation of ASBs led to a sudden decrease in flow stresses in both HTA and HTA-580 conditions, due to the thermal softening accompanied by dynamic recrystallization within the ASBs. Although the widths of ASB in HTA: 20 psi and HTA-580:20 psi were observed to be similar (~4 µm), the deformed region around the ASB was much less for HTA-580:20 psi specimen. Hence, the higher strength condition HTA-580 (precipitation strengthened condition) has narrower extend of deformation zone.
(4) The adiabatic shear localization occurs at low strains for the high strength material, and the eutectic microstructure does not delay cracking. The precipitation of L1_2_ phase in *fcc* increased the tensile strength of the alloy. However, no significant change was observed on formation of ASBs.
(5) A high density of nano-twins was observed in the region around the ASB which may be responsible for strain hardening of the alloy before ABS formation. The nano-structuring within ABS suggests dynamic recrystallization and thermal softening. A local temperature of ~800 K is estimated to have reached during testing for a few µsec causing the recrystallization in the ASBs which are adiabatically restricted in diffusion and heat flow.
(6) The microstructural characterization of the fractured specimen suggested the formation of multiple ASBs and local melting of the B2 phase and cracking within B2 phase. Pronounced recrystallization in the *fcc* phase consisting of equiaxed *fcc* grains with annealing twins were also noted. The lamellar structure seems to be broken in the region close to the fracture.
(7) Microhardness testing across the shear band showed a ~20%–30% increase in the hardness values when comparing the base hardness to the hardness in the region deformed while shear band formation. Nanoindentation was used to characterize individual lamellae before and after deformation. The hardness within the ASBs is noted to drop in all specimen.

## Figures and Tables

**Table 1:** The nanoindentation results across from HTA:20 psi, HTA-580:20 psi and HTA 580:30 psi conditions. All hardness values are in GPa.

Nano Indentation Hardness (GPa)					
Sample Condition	*fcc*		B2		In ASB
	Base	Near ASB	Base	Near ASB	
HTA:20 psi	4.1 ± 0.4	3.5 ± 1.1	6.1 ± 0.9	4.1 ± 1.2	3.4 ± 1.1
HTA-580:20 psi	5.2 ± 0.4	4.4 ± 0.2	6.9 ± 0.8	5.6 ± 0.4	3.7 ± 0.4
HTA:30 psi	4.2 ± 0.6	5.2 ± 0.7	6.0 ± 0.4	6.5 ± 1.0	5.9 ± 1.1

## Data Availability

Data will we available with the corresponding authors and can be accessed on a reasonable request.

## Supplementary Materials

The following are available online at https://www.mdpi.com/1099-4300/22/4/431/s1, Figure S1: HAADF STEM image and EDS maps from the HTA condition showing the compositional partitioning in a region with a magnified view of fcc and B2 phases. The dark contrast phase in the STEM image is B2 phase (over all rich in Al and Ni), high density nano-scale bcc precipitates can be clearly seen in the Cr map shown in blue color, Figure S2: HAADF STEM image and EDS maps from the HTA condition showing the compositional partitioning in a region with a magnified view of fcc and B2 phases. The grey/bright-contrast phase in the STEM image is fcc phase which is rich in Co, Cr and Al. No compositional fluctuation is evident in fcc phase in this heat treatment condition of the alloy, Figure S3: HAADF STEM image and EDS maps from the HTA-580 condition showing the compositional partitioning in a region with a magnified view of fcc phase. The compositional fluctuations are evidently seen in fcc phase in STEM image (bright and dark contrast). The Al-Ni rich regions correspond to the L12 phase formed in this condition after the heat treatment at 580 oC for 24 h, Figure S4: SEM image from the fractured HTA:40 psi sample. Note that the cracks are limited in the dark contrast B2 phase and are broadened to accommodate the plastic flow in fcc phase, Figure S5: Curve fitting using the equation σ=kεn (power law) for true stress-strain curve for HTA and HTA-580 condition.
