# Supplementary material for: Dynamic Shear Deformation of a Precipitation Hardened Al0.7CoCrFeNi Eutectic High-Entropy Alloy Using Hat-Shaped Specimen Geometry
Source: Entropy (Basel). 2020 Apr 10;22(4):431. doi: 10.3390/e22040431 (PMC7516913; doi:10.3390/e22040431)
Supplement: Supplementary file 1 [file entropy-22-00431-s001.pdf]

Dynamic shear deformation of a precipitation hardened $Al_{0.7}CoCrFeNi$ eutectic high-entropy alloy using hat-shaped specimen geometry

Bharat Gwalani[1], Tianhao Wang[1], Abhinav Jagetia[1], Sindhura Gangireddy[1,], Saideep Muskeri[1], Sundeep Mukherjee[1], J. T. Lloyd[3] , Rajarshi Banerjee[1,2], Rajiv S. Mishra[1,2]

[1]Department of Materials Science and Engineering, University of North Texas, Denton, TX 76207 USA

[2]Advanced Materials and Manufacturing Processes Institute, University of North Texas, Denton, TX 76207 USA

[3]U.S. Army Research Laboratory, Aberdeen Proving Ground, MD, 21005, US

Corresponding Authors: **bharatgwalani@my.unt.edu**; **rajiv.mishra@unt.edu**

Supplementary Information

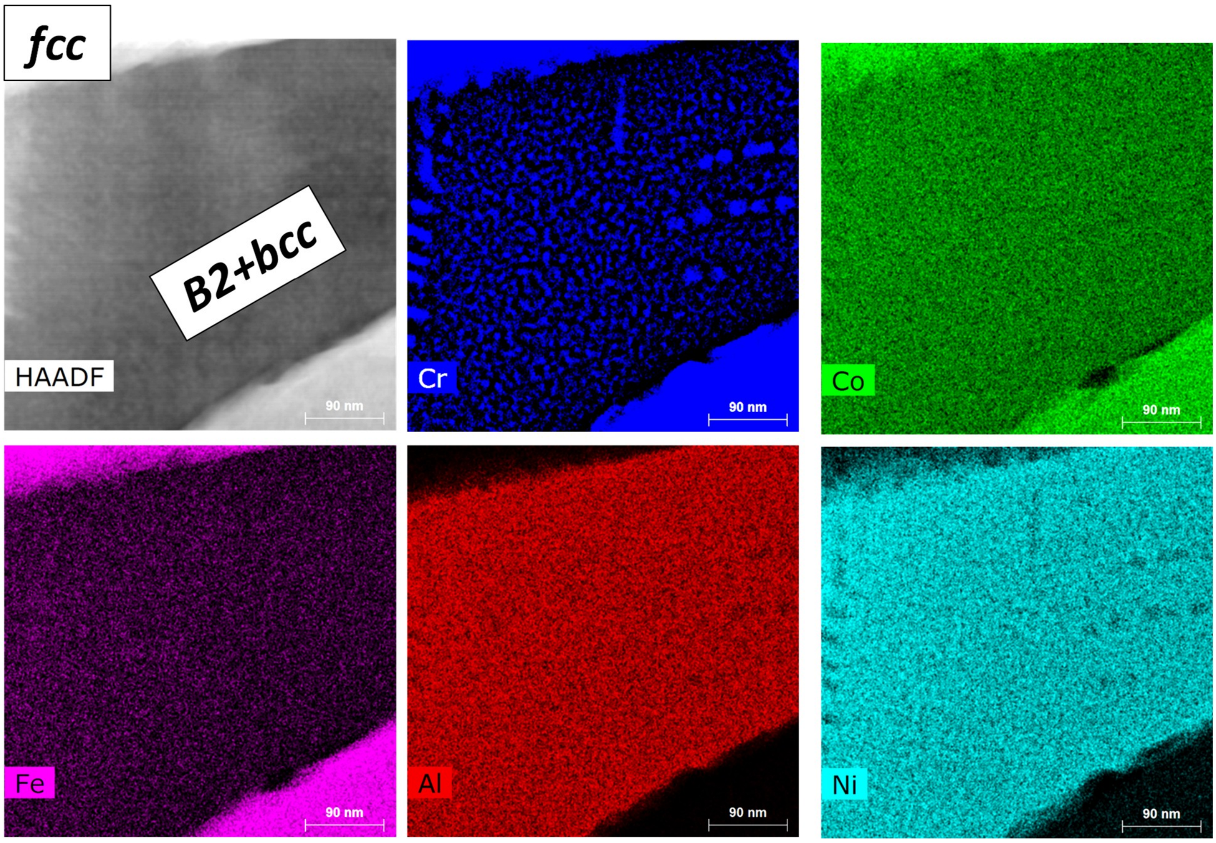

Supplementary Figure 1

HAADF STEM image and EDS maps from the HTA condition showing the compositional partitioning in a region with a magnified view of *fcc* and B2 phases. The dark contrast phase in the STEM image is B2 phase (over all rich in Al and Ni), high density nano-scale bcc precipitates can be clearly seen in the Cr map shown in blue color.

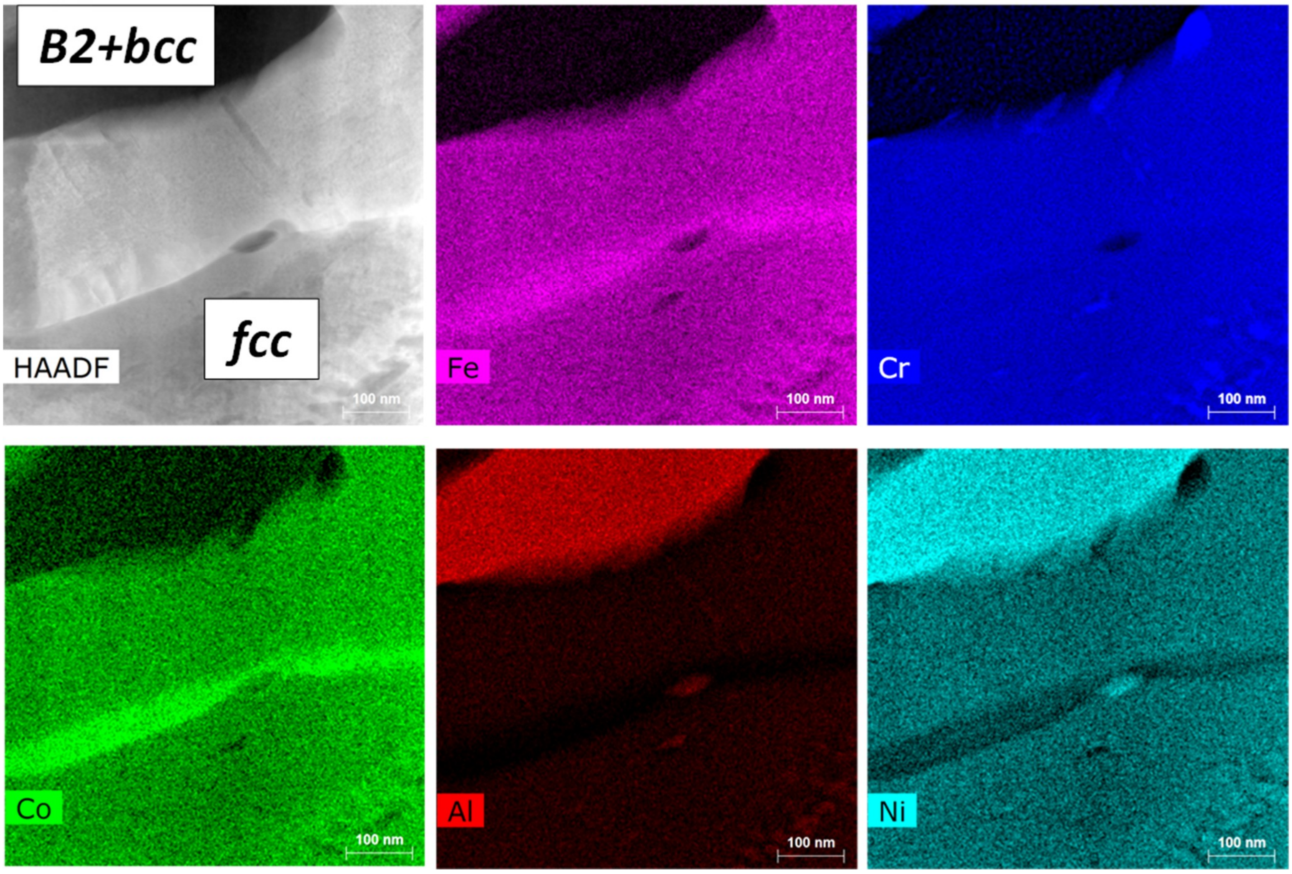

Supplementary Figure 2

HAADF STEM image and EDS maps from the HTA condition showing the compositional partitioning in a region with a magnified view of *fcc* and B2 phases. The grey/bright-contrast phase in the STEM image is *fcc* phase which is rich in Co, Cr and Al. No compositional fluctuation is evident in *fcc* phase in this heat treatment condition of the alloy.

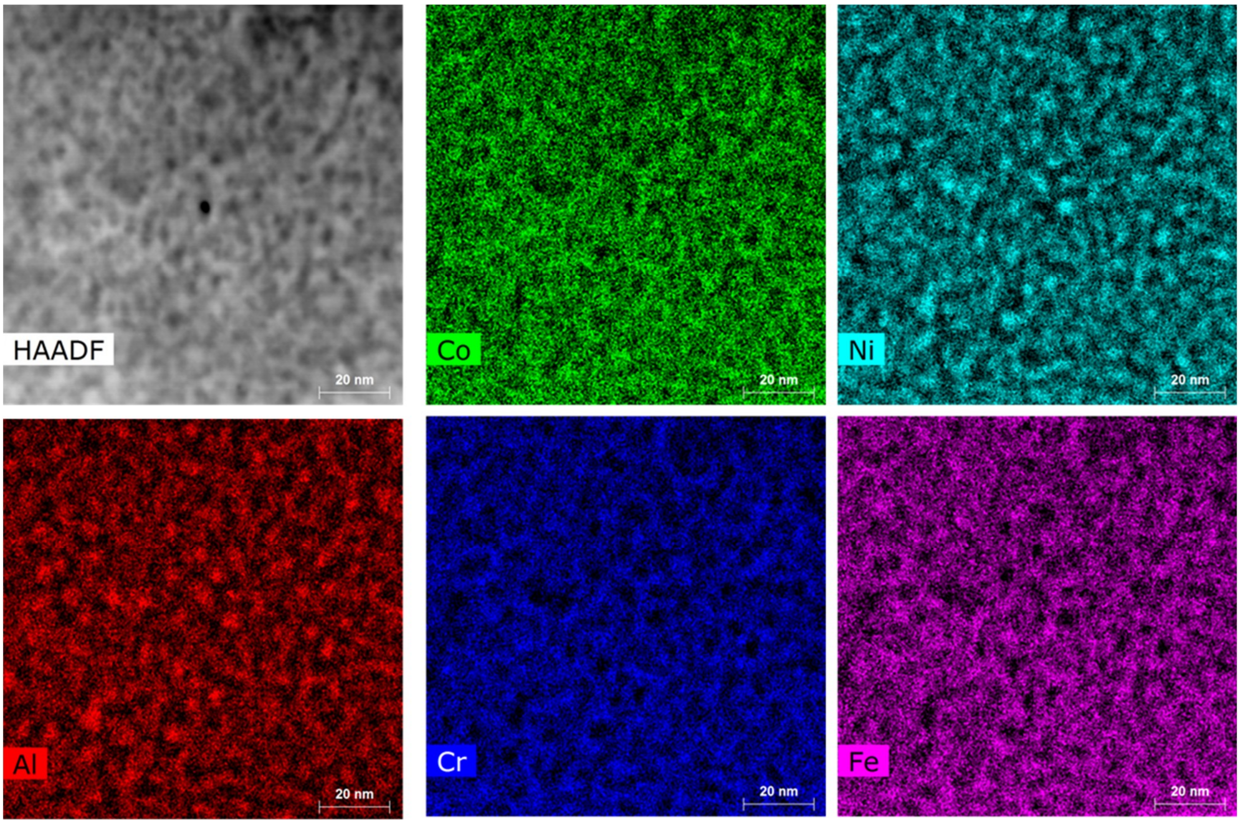

Supplementary Figure 3

HAADF STEM image and EDS maps from the HTA-580 condition showing the compositional partitioning in a region with a magnified view of *fcc* phase. The compositional fluctuations are evidently seen in *fcc* phase in STEM image (bright and dark contrast). The Al-Ni rich regions correspond to the $L1_2$ phase formed in this condition after the heat treatment at 580 °C for 24 h.

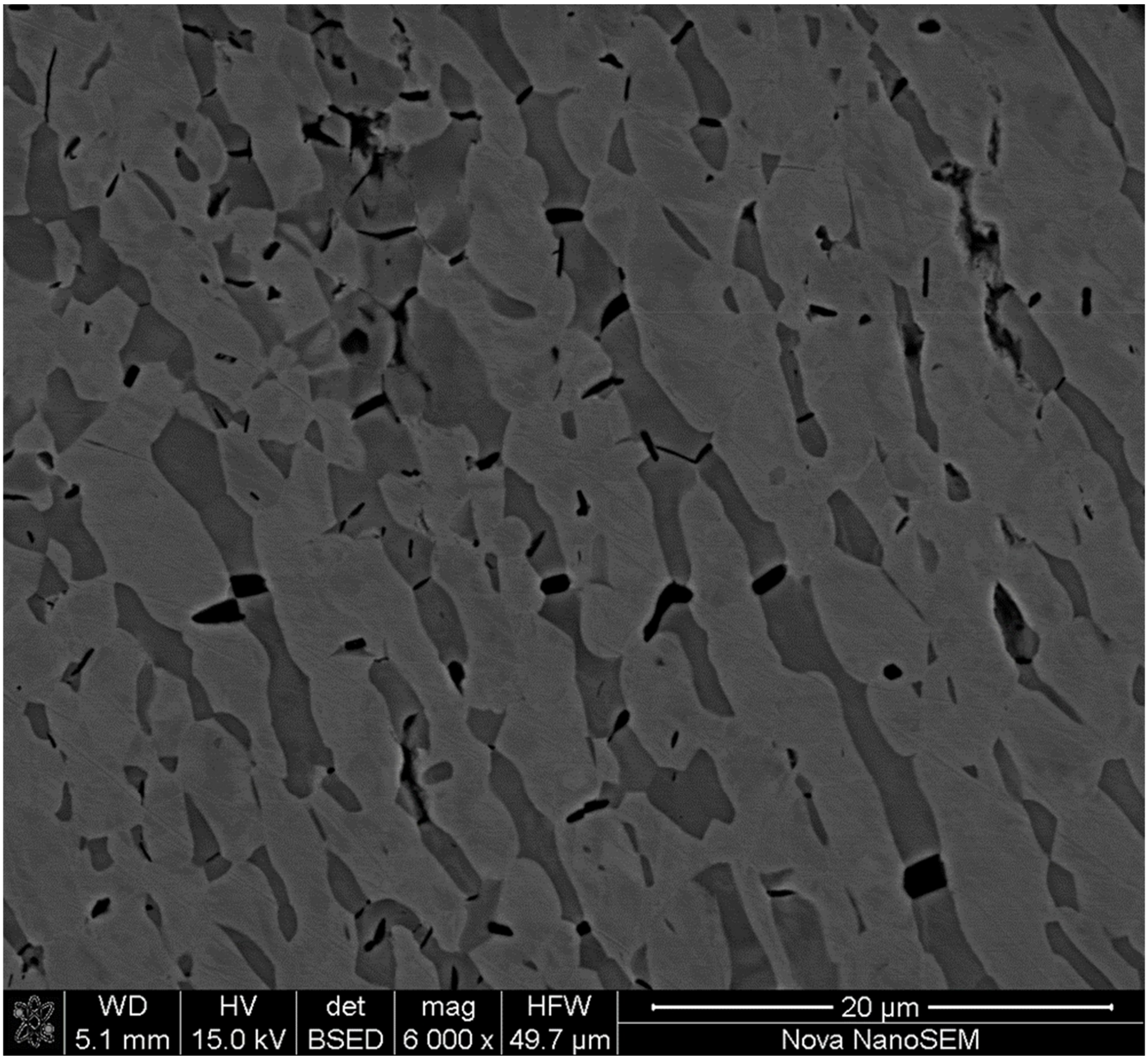

Supplementary Figure 4

SEM image from the fractured HTA:40psi sample. Note that the cracks are limited in the dark contrast B2 phase and are broadened to accommodate the plastic flow in fcc phase.

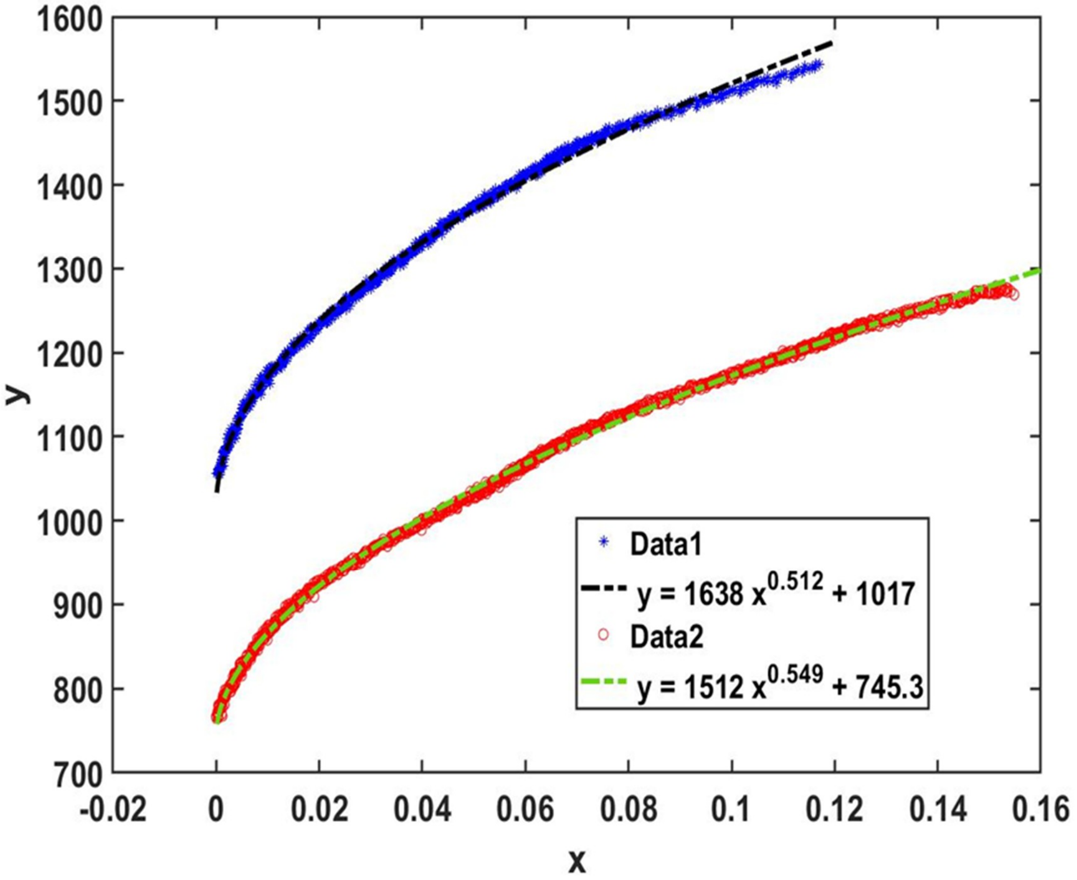

Supplementary Figure 5

Curve fitting using the equation $\sigma = k\varepsilon^n$ (power law) for true stress-strain curve for HTA and HTA-580 condition.
